# Understanding Kindness in the Russian Context

**DOI:** 10.11621/pir.2022.0105

**Published:** 2022-03-30

**Authors:** Anna V. Leybina, Mergalyas M. Kashapov

**Affiliations:** a Lomonosov Moscow State University, Moscow, Russia; b Yaroslavl Demidov State University, Yaroslavl, Russia

**Keywords:** Acts of kindness, kind behavior, kindness, network of kindness, prosocial behavior, well-being

## Abstract

**Background:**

Kindness and acts of kindness have the potential to cause tremendously positive effects on subjective well-being, reflected in improvements in mental and physical health, and interpersonal relationships. Fostering knowledge about kindness may help in self-development and psychotherapeutic interventions aimed to improve an individual’s emotional well-being. However, existing research data and understanding of this phenomenon in Russia, as well as descriptions of acts of kindness, are presently relatively limited.

**Objective:**

To study the Russian understanding of kindness, its meaning in the Russian context; to categorize a variety of identified acts of kindness; and to define kindness based on the data derived from a Russian sample.

**Design:**

There were 291 Russian participants, recruited using an online recruiting platform, who filled out an online questionnaire that identified definitions of kindness with corresponding examples. Also captured in the sample were the participant’s age, gender, and religiosity. The data underwent qualitative analysis through open, axial, and focused coding.

**Results:**

As a result of qualitative analysis, four theme categories emerged to define kindness: a) personal states and qualities (one’s own states and self-perception, moral values and qualities, self-regulation and emotional stability); b) openness to others (attention to others, love and positive attitude); c) emotional and cognitive understanding of others and tolerance, actions and behavior (altruistic sacrifice, help, politeness and respect, forgiveness, generosity, pleasing actions). Concrete examples of kind acts and behavior were categorized. A definition of kindness was formulated based on the data.

**Conclusion:**

The research results can be used in training, counselling, and therapeutic sessions to increase subjective well-being. Directions for further research have been defined.

## Introduction

Because kindness has the potential to cause such a positive effect on several important dimensions of life, the topic of kindness has been considered since ancient times. Kindness has been explored in different guises from Aristotle to Darwin (Price, 1989; Seppälä et al., 2017) and continues to be a concern to scholars. Recent studies have demonstrated that kindness improves interpersonal communication and subjective well-being (Algoe et al., 2008; Curry et al., 2018; Hui et al., 2020; Layous, et al., 2012; Shin et al., 2020); acts of kindness performed on a regular basis are documented to activate happiness-neurotransmitters or those parts of the brain associated with well-being (Harbaugh et al., 2007); overall life satisfaction and optimism increase, while anxiety and negative emotions fade (Kerr et al., 2015; Nelson et al., 2016). Moreover, the effect of kindness on longevity is widely established (Brown et al., 2009; Nelson-Coffey et al., 2017), as regular acts of kindness decrease stress (Raposa et al., 2016), pain (Emmons, 2007), and the speed of the aging processes (Hoge et al., 2013; Kok et al., 2013) for the person who performs them. Finally, there is the positive effect experienced by the recipients of kind actions (Alvis et al., 2020).

Many researchers in psychology and related disciplines are inclined to consider kindness as a separate phenomenon manifested in interpersonal communication and interaction, to be developed for the sake of the individual and societal wellbeing, as kindness and the desire to be kind to others become contagious within a community (Chancellor et al., 2018).

Scientists have defined kindness as a quality reflected within the definitions of altruism, compassion, courtesy, empathy, friendliness, generosity, love, mercy, pro-sociality, responsiveness, tolerance, intentions to do good towards others, to help in difficult situations, and to care for others (Brazhnikova & Zyuzya, 2011; Mordovina, 2014; Peterson and Seligman, 2004; Seppälä et al., 2017; Yagil, 2015). Kindness is perceived to have moral, emotional, motivational, and behavioral components (Kerr et al., 2015; Mordovina, 2014).

Knafo & Israel (2012) talk about a “network of kindness”, which includes interrelated phenomena: altruism, compassion, empathy, generosity, prosocial/helping behavior, and sympathy. Where altruism, despite significant variations in definitions, is often described as actions intended to benefit others, sacrificing one’s own interests, or performed secretly for unselfish reasons (Binfet & Passmore, 2017; Kerr et al., 2004; Yigit & Acar, 2020), compassion is an ability to sense others’ suffering and a desire to relieve this suffering (Gilbert at al., 2019); empathy is an ability to feel and understand others’ states and emotions in particular situations (Ermolova, 2016); generosity is an ability to share material and moral resources with others (Park et al., 2017); prosocial/helping behavior represents the entire spectrum of actions intended to benefit others (Torstveit et al., 2016); sympathy is an ability to relate to another’s emotions, needs, and suffering, an attempt to imagine how others feel in particular situations based on one’s own experience (Baldner et al., 2020; Nichols, 2001).

In light of the mixed definitions and limited research on kindness in the Russian context, our first research question is: What do Russians perceive kindness to mean? And by extension: Is it possible to establish a definition of kindness based on Russian respondents’ opinions?

In some sources, kindness is considered a virtue (Malti, 2020), a moral quality (Brazhnikova & Zyuzya, 2011), a character trait (Lefevor & Fowers, 2016), or a temperamental trait (Knafo & Israel, 2012). Hence, our second research question is: What is kindness in terms of a range of personality characteristics?

Kindness is discussed within certain branches of psychology and psychological theories, such as the following:

Personality psychology, where kindness is considered an aspect of agreeableness (Thielmann & Hilbig, 2015). However, irrespective of the level of agreeableness, it was found that people benefit from loving-kindness meditation and doing kind things to others (Mongrain et al., 2018).

Social psychology is generally concerned about compassion and helping behavior in diverse settings (Seppälä et al., 2017).

Positive psychology defines kindness as one of the components of character strength, “love and humanity” (Seligman, 2002), which includes the capacity for loyalty, compassion, to care about others, and to simply do good things for others on one’s own initiative (Peterson & Seligman, 2004). In particular, it is this branch of psychology that is concerned with random acts of kindness and the impact on subjective well-being (Passmore & Oades, 2015) and provides detailed recommendations for development and application of kindness in diverse settings: daily life, school, workplace (Stroekel, 2019). However, this definition of kindness is formulated predominantly through actions and behaviors: to assist, to serve, to do good, to help, to be generous, to care, to manifest compassion, altruistic love and “niceness” — where all of the above are considered as synonyms (Peterson & Seligman, 2004), which limits the definition, as it ignores internal and hidden predispositions and processes that underlie kind behavior.

Social exchange theory states that from the benefactor’s point of view, kind acts will lead to immediate or delayed positive consequences perceived as direct or vicarious reinforcement (Dufwenberg & Kirchsteiger, 2018; Honeycutt, 1981, Trivers, 1971), which can be defined under the general term, “reciprocity” (Isoni & Sugden, 2019) or “rewarding reciprocity”, which itself may have several forms (Melamed et al., 2020), including: a) direct reciprocity — a desire to pay off one’s kindness; b) reputational giving — acting kindly while being observed by people who may reinforce such behavior in various ways; c) generalized reciprocity — a kind action in response to a third party’s kindness; d) rewarding reputation — a kind act directed towards someone in recognition of their doing good to someone else. It was found that the “pay-it-forward” style of kindness has a positive impact on the well-being of both the doer and receiver (Pressman et al., 2015).

From the point of view of Poonamallee and Goltz (2014), kind actions are based on mental models that are relatively stable internal representations comprised of cognitive and emotional components based on values and attitudes. Mental models describe a person’s interaction with the world according to each unique model. All mental models involved in prosocial behavior may be divided into three categories:

1. egocentric, when kind behavior has beneficial consequences for the actor and prompt reinforcement is expected;
2. tribal-centric, when kind actions are directed toward or beneficial for a close circle of individuals or reference groups (family, friends, colleagues) and reinforcement is expected soon or in the near future;
3. transcendental, when actions are directed towards strangers or society in general, e.g., in cases of emergency (Douty, 1972). Possible benefits are expected in the remote future, if they are expected at all.

Social exchange theory often considers Kohlberg’s stages of moral development (Comunian, 1998), where the emphasis is placed on prosocial development with age.

In spite of evolutionary considerations of prosociality highlighted by the theory, internal predispositions and motivation for kind acts are viewed as rather self-serving, and kindness is considered only as helping behavior or generosity.

Theory of reasoned actions (Caldwell, 2017; Fishbein & Ajzen, 2010) defines kindness mostly through kind actions that integrate cognition, attitudes, and intentions resulting from beneficence, a character trait or virtue which is described though altruism, compassion, generosity, mercy, humanity, and love. This theory, in the context of kindness and its inherent nature, describes kindness from the standpoint of several core beliefs, attitudes, intentions, and behaviors.

- *Beliefs* refer to: behavior (what is expected from oneself and others, understanding of others’ needs), normative (moral beliefs and judgments regarding acceptable behavior), control (beliefs about own capability to behave in a right way).
- *Attitudes* refer to: behavioral values (self-image and identity; perception of one’s own compassion and empathy), perceived norms (behavior that would be ethical and appropriate), perceived control (the degree of confidence that one’s actions that will make a difference)
- *Intentions* refer to: benevolence that will allow beliefs and attitudes to be manifested in kind actions; willingness to act upon an assessment of needed efforts and expected results.
- *Behavior* is simply the way a given kind act is actually carried out.

This theory, while useful in describing several internal predispositions and sources of kind actions, does not provide a clear definition of kindness, or illustrations and categorization of kind behavior.

### Activity theory

The main components of this theory are activity, consciousness, and personality. Activity is a goal-oriented behavior which has several levels of consideration: special activity, actions, operations, and psychophysiological functions (Leontiev, 1981). Activity is comprised of actions that can be executed in various ways—operations that depend on conditions and contexts. For example, the conditions and context of a given situation will determine what type of kindness will be extended. Goals and motives are at the basis of every activity (Leontiev, 1981). Derived from pedagogical kindness research, this theory provides an understanding of kindness (Leybina et al., 2020). The following themes in the definition of kindness have been identified by research conducted with Russian teachers:

- personal states and qualities: one’s own states, values, and moral characteristics, self-regulation and emotional stability;
- openness to others: attention to others, love, and a positive attitude;
- tolerance and cognitive and emotional understanding of others;
- external actions and behavior: help, courtesy and respect, kind and pleasant actions;
- internal actions and behaviors: forgiveness, altruistic internal actions.

Activity theory can be applied here: kindness is not simply a reaction to the environment (e.g., to others’ problems), but a well-intended process generated also from within the individual.

It is possible to see some similarities here with the theory of reasoned actions. Activity theory provides meaningful background to study goals and motives in terms of what constitutes kind behavior, and to research the mechanics of corresponding kind actions; these issues currently require further investigation.

Obviously, these theories serve diverse yet important purposes in kindness research. In particular, social exchange theory may be used to study the rationale behind kind behavior and to assess the benefits for those who act kindly. The theory of reasoned actions will allow a study of internal predispositions (beliefs, attitudes) and transformations of kind intentions into kind actions. Activity theory may allow for an assessment of complex reasons, goals, and motives for kind actions, as well as analysis of the operations driving kind actions of a particular type. Additionally, activity theory contributes to investigation of kind acts: decision making, execution, control, and correction. Also, the theory deals with both internal and external actions. All of the above speak in favor of an integrative approach that combines several of these theories to study kindness phenomena in the context of the two central questions addressed herein.

It can be surmised at this stage that all the approaches and theories outlined above indicate that the external manifestations of kindness are indicators of personal qualities. The degree of kindness needs to be evaluated by the intended recipient (Binfet et al., 2016), as a seemingly kind act may be considered unkind, in some cases, by the person it is intended for. It is the way the intentions behind such acts are perceived that is the salient point; for example, if the intentions are hypothesized to be kind, the act itself will be considered kind, regardless of the results (Falk & Fischbacher, 2006).

The range of kind actions is extremely diverse and depends significantly on the situational and cultural context (Layous 2013; Shin et al., 2020). Kind actions lead to an increase in well-being (Curry et al., 2018; Stroekel, 2019). Kind behavior is widely promoted across countries and societies; however the behaviors selected for promotion are of particular relevance. Due to increases in well-being and mental health, a diverse range of kind behaviors has been suggested by scientists, practitioners, and foundations (e.g., Mental Health Foundation, 2020). Individuals choose and practice acts that would be appropriate for their given situation.

It has been demonstrated that memories of kind actions may be as beneficial as the kind acts themselves, both for the person who performs them and for the recipient (Exline et al., 2012; Ko et al., 2019). Hence, a precise classification of kind acts would allow questionnaires for therapeutic intervention that may lead to positive mental health and transformation effects.

However, despite several attempts, precise categorization of kind acts has still not been achieved. Canter et al. (2017b) provided classification for different modes of kindness:

- psychologically passive kindness, which does not require any active behavior (e.g., to let a person who is in a hurry cut into a queue);
- principled proaction, which assumes active help (e.g., volunteering, donations);
- affective-psychologically passive kindness, which is grounded in social norms (e.g., to help when asked; to help some one pick up dropped belongings), which also includes an emotional component (e.g., to allow a person to talk about their problems, to listen);
- affective-proactive kindness (e.g., to secretly search for a gift that can make another person happy; to cancel a trip to stay with a friend or relative who is in need).

Research on this classification is still in progress. Moreover, the data was gathered predominantly from British or English-speaking samples. Although this concept has been found somewhat relevant for Russian teachers (Leybina et al., 2020), the data for Russians may differ to a meaningful degree.

Another aspect of significance is the range of kind behavior, which narrows down a limited number of activities. This focused set of activities can be used in well-being development programs based on kindness. Hence, the third research question is as follows: Is it possible to provide a clear classification of kind actions based on a Russian sample?

Considering the three research questions, the aim of the current research was to study how Russians understand kindness, to categorize acts of kindness, and to define kindness based on data from a Russian sample.

## Methods

### Participants

There were 291 Russian participants, who currently reside in Russia. Several studies provide evidence that kindness and kindness perception are influenced by socio-demographic characteristics: age and gender (Canter et al., 2017a; Hui et al., 2020), and religiosity (Arslantürk & Harupt, 2020; Bekkers et al., 2020); hence these were used to describe the current sample. The sample comprised 152 (52.23%) females and 139 (47.76%) males. *Table 1* contains the information on socio-demographic characteristics of the participants depending on gender.

**Table 1:** Socio-demographic characteristics based on gender

Variable	Male	Female
Age	*M* = 38.52 (*SD* = 9.11)	*M* = 37.63 (*SD* = 10.47)
Religiosity		
Yes	59 (20.27%)	55 (18.90%)
No	80 (27.49%)	97 (33.33%)

### Measures

The web-based questionnaire was placed on participants’ recruiting platforms. The participants were asked to specify their age, gender, and religiosity, to define kindness from their point of view, and to provide examples of kind acts.

### Procedure

Data analysis was performed using Intellectus Statistics (2020) for quantitative analysis; qualitative analysis was executed via QDA Miner Lite v 2.0.8. (Provalis Research, 2020) in the form of standard content analysis.

An open coding procedure was used to analyze participant responses based on definitions and examples of kindness (Charmaz, 2014), which resulted in theme classification (Ryan & Bernard, 2003); codes were assigned to these themes for categorization. Subsequently, axial coding was executed (Charmaz, 2014) to group the generated themes into their respective categories. In cases where a response contained more than one theme, these themes were assigned different codes and considered separately. Similar theme categories were grouped into larger categories. Focused coding was used to divide a set of exemplary kind actions in accordance with previously identified themes of kind actions and behaviors (Charmaz, 2014). All the data were collected and processed in the Russian language. English translations of participants’ responses are provided in the present article.

## Results

### What is kindness from a Russian point of view?

When asked to define kindness 96.91% of respondents provided specific “codable” answers (n = 621). From these answers, 14 themes emerged, which are possible to group into four categories (Table 2).

**Table 2:** Coding manual for kindness definition themes

Theme	Answers (%)	Example descriptions
Personal states and qualities	12.88	General internal predispositions related to attitudes towards oneself and the world, values and moral characteristics, and ability for self-regulation
1. One’s own inner states	6.12	“To be honest with oneself ”, “to see the world from bigger perspective”, “to concentrate on good things”, “positive thinking”, “internal light”, “self-respect”, “love yourself ”, “happiness”, “positivity”, “to live in harmony with the world”, “internal harmony”, “internal warmth”, “internal strength”
2. Values and moral characteristics	5.31	“Justice”, “sincerity”, “honesty”, “responsiveness”, “peacefulness”, “golden rule: to do to others what you want for yourself ”, “reliability”, “conscientiousness”, “trust”, “mercy”
3. Self-regulation/ emotional stability	1.45	“Emotional adequacy”, “to be calm and to demonstrate positive emotions”, “ability for emotional self-regulation”, “calmness”, “mental fortitude”, “equilibrium”, “patience”
Openness to others	19.32	Attention to others and general positive attitudes towards others
4. Attention to others	9.50	“Responsiveness”, “open heartedness”, “disposition to people”, “attention to people”, “hospitality”, “involvement”, “to think about others”, “sensitivity”
5. Love and a good attitude	9.82	“To love people”, “to see good in people”, “liking”, “humanity”, “careful attitude towards others”, “positive attitudes for people in general”, “good opinion about everyone”
Understanding others	21.09	“To place oneself in others’ shoes”
6. Understanding others with the mind	4.83	“To understand people”, “try to understand others”
7. Understanding the emotions of others	10.95	“To understand others’ feelings and emotions”, “empathy”, “compassion”, “sympathy”, “mercy”
8. Tolerance	5.31	“To accept others’ peculiarities”, “to accept others’ opinions, different from yours”, “to accept people the way they are with all their flaws”, “not to judge others”, “loyalty”, “tolerance”, “leniency”, “acceptance of others’ weaknesses”
Actions and behaviors	46.71	Observed or hidden actions and behaviors intended to cause positive influence on others and others’ lives
9. Help	23.18	“To assist in difficult situations”, “support”, “care”, “mutual help”, “to listen when needed”
10. Politeness/ respect	4.34	“Respect”, “smile”, “niceness”, “gratitude”, ‘tenderness”, “to say something good about somebody”
11. Pleasing action	5.15	“Participation in others’ happiness”, “to make others happy”, “to do useful things”, “nice gestures”, “positive actions towards others”, “to make others feel comfortable”, “to create friendly atmosphere”
12. Generosity	1.93	“To share happiness and positivity”, “to share resources”, “material support”, “charity”
13. Forgiveness	1.93	“To forgive an enemy”, “not to hold grudges”, “to forgive mistakes”
14. Altruistic self-sacrifice	10.18	“Concessions”, “self-sacrifice”, “dedication”, “unselfishness”, “not to expect anything in return”, “to sacrifice a resource for the sake of others”, “to give to others more than to yourself ”, “to do good despite inner obstacles”

*Note: respondents’ words were used (translated from Russian)*

It is clear that most of the definitions are related to actions and behaviors (46.71%), which falls in line with the positions of the above-mentioned theories and approaches, and includes all the components of the “network of kindness” (empathy, compassion, sympathy), as well as understanding others (21.09%). However, 32.2% of the definitions refer to inner aspects or external manifestations of internal states. Hence, it is reasonable to say that a narrow concentration only on kind behavior, or the “network of kindness”, may limit a fuller insight into the study of kindness phenomena.

### Examples of kind actions

When asked to provide examples of kind acts, 97.60% of respondents gave specific “codable” answers (n = 558). These were categorized in accordance with the themes identified above (Table 3). In accordance with previously discussed theories, it is hypothesized that the defined states and qualities, such as openness to others and an ability to understand others, will be manifest through internal or external actions. All participants who provided examples referred to the set of kind acts and behaviors identified earlier (themes 9–14) and not to themes 1–8, which suggests that the later themes refer to internal predispositions toward kind behavior.

**Table 3:** Examples of kind acts

Theme	Answers (%)	Examples
9. Help	54.65	“To listen to a person if he/she feels bad”, “to help”, “to assist the elderly in crossing the street”, “to do some work in the garden for parents”, “to relieve suffering”, “to join someone in their work so the person finishes it faster”, “to look after an ill person”, “to help carry heavy groceries”, “volunteering”, “to help in spatial orientation and navigation”, “to help in something you are good at”, “to save an animal”, “rescue”
10. Politeness/ respect	4.66	“To hold the door for a stranger”, “to give up your seat in a public transport”, “to calm down a rude person”, “to try to understand and acknowledge the position of another person”, “to stay polite despite rudeness”, “to show respect and a good attitude”, “to withhold your opinion”, “to restrain one’s own words and emotions”
11. Pleasing actions	8.60	“To ask about another’s state”, “to call and talk about how things are going”, “to surprise someone with a present”, “to give sweets just to make another person happy”, “to compliment a stranger to make them smile”, “to cheer someone up”, “to find a client for somebody’s business”, “to invite a retired person to talk with young people to make him/her feel needed”, “to recommend a good job vacancy to a friend”, “to draw a picture or to create another piece of art to make others happy”
12. Generosity	20.07	“To give something away”, “to buy things needed for others”, “to feed a homeless person”, “to share surplus harvest from your garden”, “to share internal warmth”, “to donate money”, “to provide financial help”, “to spend time with someone”, “to give away old things”, “to bake a cake and to share it with a neighbor”, “to be a donor”
13. Forgiveness	3.94	“One person let another person down, and the later did not get offended but asked if he/she can be of any help”, “to forgive and let go”, “not to respond to aggression with aggression”, “to forgive a person who abused ones’trust”
14. Altruistic self-sacrifice	8.08	“To listen to another person when not interested”, “to give your ice cream to a child if he/she dropped theirs”, “to assist a stranger even if it means being late to an important meeting”, “to secretly help”

*Note: respondents’ words were used (translated from Russian)*

Based on the sample results, it is possible to define several tendencies:

1. Help (54.65%) and generosity (20.07%) received the most examples, consistent with social exchange theory. Other themes also received corresponding examples from the sample, which indicates the need for more precise categorization of kind acts. There were no examples that could not be categorized.
2. Among behaviors that were categorized as “help”, a special group of examples identified as “rescue” gathered 9% of the examples, mostly referring to animals.
3. Some actions may be both internal and external. For example, it is possible to forgive internally, or to openly demonstrate forgiveness.
4. Some categories of kind actions are interrelated, so it is impossible to identify them as a single type (e.g., “material assistance” may be considered as help, and as generosity).
5. Withholding negativity is also considered as an act of kindness: “to keep one’s opinion for oneself ”, “to restrain one’s own words and emotions”, “not to respond to aggression with aggression”.
6. The examples provided by Russian respondents reflect kindness behaviors identified by Canter et al. (2017b) including: psychologically passive kindness — “to demonstrate respect”, principled proaction — “volunteering”, “donation”, affective-psychologically passive kindness — “to assist in spatial orientation”, “to listen to others when needed”, and affective-proactive kindness — “to help while keeping it a secret”. There were, however, actions that are impossible to categorize based on the perspectives of Canter and colleagues, especially those that refer to pleasing actions, politeness, respect, and forgiveness.
7. There were no specific examples relating to sympathy, empathy, or compassion, which may suggest that these characteristics should be regarded as internal predispositions of kind acts, even though they can be expressed and observed.

## Discussion

This discussion aims to answer the four research questions formulated in the Introduction.

### How kindness is understood by Russians

Based on the qualitative analysis of the respondents and their answers, the following themes and theme categories have been identified:

#### Theme category 1

Personal states and qualities: one’s own inner state, values and moral characteristics, self-regulation and emotional stability. This theme category refers to the set of inner qualities mentioned by respondents as kindness. It is assumed that these characteristics can be considered as separate psychological phenomena, e.g., values or self-regulation, and their exact connection with kindness is yet to be explored.

#### Theme category 2

Openness to others: attention to others, love and a good attitude. This theme category refers to orientation towards others, and, again, can be considered as a set of well-known psychological phenomena.

#### Theme category 3

Understanding others: understanding with the mind, understanding the emotions of others, tolerance. This theme category essentially refers to characteristics usually attributed to empathy and sympathy as essential components of kindness.

#### Theme category 4

Actions and behaviors: help, courtesy and respect, pleasing actions, generosity, forgiveness, altruistic self-sacrifice. All of the sub-categories have been considered by various of the abovementioned authors as kind.

Evidence from the sample indicates that from the Russian point of view, kindness can not only be described in terms of activity and behavior, but also includes internal attitudes and predispositions. It is also evident that the Russian perspective is not limited to the phenomena included in the “network of kindness”, because it is not restricted to definitions of altruism, compassion, empathy, generosity, prosociality, and sympathy, but rather also includes other characteristics. Responses from this Russian population in general is not significantly different from that of Russian educators (Leybina et al., 2020), which is reasonable, since Russian teachers also represent the Russian population. There are, however, several differences. Generosity was not mentioned by the Russian teachers, but was highlighted by 1.93% of the participants in the current research, which goes in line with other studies. Altruistic sacrifice has been defined as a more precise category (vs. altruistic behavior for educators), where the accent has been made on self-sacrificing behaviors to benefit others. Also, for the Russian population in general, unlike for educators, it was not possible to provide a clear differentiation for internal and external kind actions, as respondents’ definitions of kindness referred to actions and behaviors that can equally be hidden and observed. The reasons for such differences are yet to be explored.

### Kindness as a personality characteristic

Based on participant responses, kindness is understood as a set of internal predispositions, characteristics, and abilities that are reflected in actions and behaviors considered as kind. Values and moral characteristics are considered as the ingredients of kindness, but do not provide a full explanation of this phenomenon; rather they describe internal sources of kind behavior. Despite convincing arguments provided by Knafo and Israel (2012) regarding kindness being a temperamental trait, their conclusion is based on narrow research relating to the separate elements that comprise a “network of kindness”. Temperamental traits may contribute to the ability to self-regulate and be open to others (Strelau, 2020; Trofimova et al., 2018), but given the current findings, kindness cannot simply be called a temperamental trait. However, considering the evident connection between character and temperament (Asmolov, 2001), as well as definitions of character (Borozdina, 2015; Gippenreiter, 2020) as one of the personality’s substructures, which comprises definitions of the “stable individual” (i.e., traits and predispositions), what emerges through the analysis of individuals in the context of social interactions is a definition of kindness as a set of character traits which are closely aligned with the definitions of agreeableness outlined by scholars of personality psychology (Mongrain et al., 2018).

Therefore, kindness in the Russian context can be defined as a character trait generated by personal states and qualities, openness to and ability to understand others, which is manifested in external and internal positive actions and behaviors towards others.

### Classification of kind actions

Six categories of kind behavior have been revealed: help (including rescue), polite/ respectful actions, pleasing actions, generous actions, acts of forgiveness, and altruistic sacrifice. Some actions, however, cannot be unequivocally categorized and may overlap with more than one category of kind acts and behaviors. Canter and colleagues’ classification can be considered, and yet, some examples provided by the respondents do not fit into this classification. It is clear that more detailed categorization for prosocial/helping behavior is required; for example there are several forms of volunteering (Kelemen et al., 2017).

## Conclusion

The results of current research can already be used for various purposes: to create and log “a personal acts diary” (Kerr et al., 2015); to enhance kindness for well-being purposes by accessing memories of kind acts conducted or experienced in the past (Exline et al., 2012); to develop the ability and inclination to compliment others (Boothby & Bohns, 2020); to develop detailed questionnaires designed to assess kindness levels for purposes of providing directions for personal development; and to construct thematic training programs in order to achieve positive psychological transformation. For future research, it is recommended to analyze goals and motives that elicit kind behavior; to define the mechanics of kind behavior (actions and operations); and to study the ingredients that lead to kind behaviors: decision making, execution, control, and correction. It may also be possible to consider kindness as a resource quality. Quantitative measures to explore the current construct can also be suggested.

## Limitations

First, given the limitations of sampling method, we are unable to assess the response rate and drop-out rate. Therefore, a significant limitation of our work lies in the possibility that the results reflect self-selection along dimensions relevant to kindness— put simply, more altruistic individuals may have been more likely to participate, distorting perception of kindness and biasing our results in the direction of greater prosociality than is actually prevalent in the general Russian population.

Second, given that participants knew that the survey was about kindness, their responses might have been affected by social desirability bias (Paulhus, 1984).

Third, because this was a cross-sectional design, recent experiences, transient affective states, and other variable factors may have influenced participants’ responses.

Despite the above limitations, our study benefited from a sample that was substantial in both size and diversity along the measured demographic variables, allowing us to present a preliminary portrait of kindness as it is perceived by Russians. We hope that our findings will be of use as scholars continue to probe the endogenous and exogenous determinants of kindness, that most vital of human characteristics.
